# Extracted step parameters during the timed up and go test discriminate between groups with different levels of cognitive ability—a cross-sectional study

**DOI:** 10.1186/s12877-025-05828-6

**Published:** 2025-03-17

**Authors:** Niklas Löfgren, Lars Berglund, Vilmantas Giedraitis, Kjartan Halvorsen, Erik Rosendahl, Kevin J. McKee, Anna Cristina Åberg

**Affiliations:** 1 School of Health and Welfare, Dalarna University, Falun, 79 182 Sweden; 2 Department of Public Health and Caring Sciences, Geriatrics, Uppsala University, Box 564, Uppsala, SE-751 22 Sweden; 3 Epistat AB, Dag Hammarskjölds Väg 10C, Uppsala, 752 37 Sweden; 4 Department of Community Medicine and Rehabilitation, Physiotherapy, Umeå University, Umeå, Sweden; 5 CIRCLE - Complex Intervention Research in Health and Care, Department of Women’s and Children’s Health, Uppsala University, Uppsala, Sweden

**Keywords:** Motor-cognitive dual-task, Gait, Dementia, Mild cognitive impairment, Subjective cognitive impairment, Cognitive decline

## Abstract

**Background:**

Identifying cognitive impairment at an early stage is important to enable preventive treatment and lifestyle changes. As gait deviations precede cognitive impairment, the aim of this study was to investigate if step parameters during different Timed Up and Go (TUG) conditions could discriminate between people with different cognitive ability.

**Methods:**

Participants (*N* = 304) were divided into the following groups: (1) controls, *n* = 50, mean age:73, 44% women; (2) Subjective cognitive Impairment (SCI), *n* = 71, mean age:67, 45% women; (3) Mild Cognitive Impairment (MCI), *n* = 126, mean age: 73, 42% women; and (4) dementia disorders, *n* = 57, mean age: 78, 51% women. Participants conducted TUG and two motor-cognitive TUG-conditions: TUG while naming animals (TUGdt-NA) and reciting months in reverse order (TUGdt-MB). Tests were video recorded for data extraction of valid spatiotemporal parameters: step length, step width, step duration, single step duration and double step duration. Step length was investigated with the step length/body height ratio (step length divided by body height). Logistic regression models (adjusted for age, sex and education) investigated associations between step parameters and dichotomous variables of groups adjacent in cognitive ability: dementia disorders vs. MCI, MCI vs. SCI, and SCI vs. controls. Results were presented as standardized odds ratios (sORs), with 95% confidence intervals (CI^95^) and *p*-values (significance level: *p* < 0.05). The areas under the Receiver Operating Characteristic curves were presented for the step parameters/conditions with the highest sORs and, where relevant, optimal cutoff values were calculated.

**Results:**

Step length showed greatest overall ability to significantly discriminate between adjacent groups (sOR ≤ . 67, CI^95^: .45-.99, *p* = ≤ . 047) during all group comparisons/conditions except three. The highest sOR for step-length was obtained when discriminating between SCI vs controls during TUGdt-MB (sOR = .51, CI^95^:.29- .87, *p* = .014), whereby the area under the curve was calculated (c-statistics = .700). The optimal cut-off indicated a step length of less than 32.9% (CI^95^ = 22.1–43.0) of body height to identify SCI compared with controls.

**Conclusions:**

The results indicate that step length may be important to assess during TUG, for discrimination between groups with different cognitive ability; and that the presented cut-off has potential to aid early detection of cognitive impairment.

**Trial registration number:**

NCT05893524 (retrospectively registered 08/06/23).

**Supplementary Information:**

The online version contains supplementary material available at 10.1186/s12877-025-05828-6.

## Background

Cognitive impairment refers to increased difficulties conducting everyday tasks relying upon, for example, verbal fluency, processing speed, working memory and executive functions [1]. This condition becomes more prevalent with increasing age, ranging from subjective cognitive impairment (SCI) to more moderate or severe impairment, in the forms of mild cognitive impairment (MCI) and dementia disorders, respectively. Whereas SCI is defined as self-reported impairment that cannot be identified by standard objective cognitive assessment [2], MCI is detectable at cognitive assessment while the affected individual is still able to manage everyday tasks [3]. Nevertheless, MCI is related to decreased independence, less participation in social activities, and increased depressive symptoms [4], while up to 15% of MCI individuals receive a dementia diagnosis each year [5]. The collective term dementia disorders refers to a number of highly progressive dementia-related conditions that are the leading cause of dependency and disability among older adults [6]. Not only do such conditions have a negative effect on the person with dementia and their family, they are also costly for society [7], with dementia targeted as a public health priority by the World Health Organization. [8]. Hence, to reduce the burden of cognitive impairment, and particularly dementia, it is vital to identify early signs of impairment in order to enable preventive treatment and induce lifestyle changes that may delay disease progression [9].

In recent years the interactions between cognition and motor abilities, not least gait, have received greater recognition. Indeed, individuals with cognitive impairment have been found to walk with an altered gait pattern in comparison with age-matched counterparts when assessed during continuous overground walking [10–12]. In addition, studies have found slower gait speed to precede cognitive impairment by up to twelve years [13], whereas an abnormal gait pattern [14] as well as increased difficulties in walking with a concurrent cognitive task (i.e. motor-cognitive dual-task) have been found to indicate future conversion from MCI to dementia [15]. Together, these findings highlight the potential of gait assessment as a primary screening tool for older adults. However, most of the above results derives from assessments of gait speed, while gait is multidimensional [16]. Therefore, it is relevant to also investigate if specific step parameters can be used as a complement to gait speed in discriminating between groups with different cognitive ability. In addition, assessments have generally been conducted in motion laboratory settings (i.e. advanced equipment) [10, 12, 15] or have been conducted in clinical settings requiring spacious environments [13], which may not be available in most clinical facilities and therefore hampers potential for implementation. Also, few studies have been able to discriminate between different groups with less severe impairment (i.e. people with MCI/SCI and healthy controls) [10]. Hence, it is highly relevant to combine innovative techniques with clinically applicable tests under challenging conditions to also explore if certain step parameters have the potential to discriminate between groups with low levels of impaired cognitive function.

The Timed Up and Go (TUG) test is a widely used test that is assessed by registering the time it takes for a participant to rise from a standard chair with armrests, walk three meters, turn 180°, walk back to the chair and sit down again [17]. Hence, TUG is well suited for clinical practice as it is both space and resource efficient with the only equipment needed being a stopwatch and a standard armchair. Originally developed to assess functional mobility in older populations, TUG has been found to have adequate psychometric properties when assessed in a variety of clinical populations [18]. Moreover, as opposed to continuous overground walking, the TUG includes subphases that challenge gait adaptation and motor planning, abilities that are crucial for independent ambulation and compromised in cognitively impaired individuals [19]. TUG can also be assessed as a motor-cognitive dual-task (TUGdt), thereby challenging executive function. Accordingly, it has been suggested that motor-cognitive dual-task performance may be a more effective diagnostic tool than gait alone in people with dementia [20].

Within the Uppsala-Dalarna Dementia and Gait project (UDDGait™) [21], we have integrated the investigation of the TUG performance under single-task and two different TUGdt conditions in participants undergoing memory assessment in specialist clinics. The two TUGdt conditions were (1) naming animals (a condition related to verbal fluency) and (2) counting months in reverse order (a condition related to cognitive inhibition). In addition, in this project we have used an innovative, deep learning-based technique (previously found reliable and valid [22]), to extract potentially sensitive spatiotemporal step parameters from video recordings of the TUG performance. Previously, we have identified that the outcome number of animals/months recited per 10 s discriminates between groups with different cognitive ability [23]. For the current study, the aim was to investigate if specific spatiotemporal step parameters i.e. step length, step width, step duration, single step (SS) duration and double step (DS) duration extracted during TUG and two TUGdt conditions, were able to discriminate between groups of people with: intact cognitive ability; SCI; MCI; and dementia. If indicated by the results, a secondary aim was to investigate clinically relevant cut-off scores for applicable step parameters.

## Methods

### Design, setting and study participants

The current study had a cross-sectional design and formed part of the ongoing, longitudinal UDDGait project [21]. In UDDGait patients (*N* = 298) were recruited during 2015–17 when undergoing memory assessment at two specialist clinics in Sweden following referral from a family physician or when independently booking an appointment. The procedure for cognitive diagnosis followed established criteria [24–27], was part of the clinical routine for patients assessed for memory impairment and was conducted by a clinical geriatrician. The assessment included careful evaluation of the patient’s history, structural brain imaging, and cognitive testing. The cognitive tests included: the Clock Drawing Test, the Verbal Fluency Test, and Trail Making Test A and B. Supplemental assessments such as neuropsychological testing and cerebrospinal fluid analysis were carried out when considered relevant. Participants that complained about cognitive decline and had taken part in full cognitive assessment where clinical evaluation and objective measures did not indicate cognitive impairment, were diagnosed with SCI [28]. Exclusion criteria for patients were: inability to walk three meters back and forth or to rise from a sitting position, indoor use of a walking aid, current or recent hospitalization (within the last 2 weeks), or need of an interpreter to communicate. In addition, control participants (*N* = 166) were recruited through advertisements and flyers (May 2017 to March 2019). The inclusion criterion for controls was normal cognitive function, while exclusion criteria were the same as for the potential study patients.

In the current study we use available dual-task step parameter data from UDDGait, which were extracted from video recorded tests. First, video recordings from patients were included (*n* = 254). Then, recordings were selected from controls (*n* = 50) representative of the gender and age spectrum of the included patients. Thus, data from 304 participants were analysed. Ethical approval was granted from the Regional Ethical Review Board in Uppsala.

### Test procedures

Data collection for all participants was carried out by a trained physiotherapist and followed the procedure that has been described in more detail elsewhere [21, 29]. Demographic data were collected through self-reports from the participants and, if he/she so wished, also from a relative. Depression screening was carried out using the four-item version of the Geriatric Depression Scale (GDS-4) [30]. Tests of motor and cognitive functions were performed as follows: hand grip strength measured by a dynamometer; mobility using a short version of the General Motor Function Assessment Scale [31, 32]; balance in accordance with the Bohannon Method [33]; and different aspects of cognitive function by the Clock Drawing Test, the Trail Making Test and the Verbal Fluency Test [34].

For the TUGdt tests two verbal tasks were used: “name different animals” (TUGdt-NA) and “recite the months of the year in reverse order” (TUGdt-MB). Participants were instructed to complete both tasks at comfortable speed, and to prioritise the walking part – i.e., if he/she was unable to say words, he/she should continue walking and complete the movement sequence. All TUG tests were timed by a stopwatch from the participant’s back leaving the chair backrest to their posterior touching the seat of the chair again, and time scores for the total performance of each TUG and TUGdt test were noted. The tests were also recorded by two synchronized high-definition video cameras. The test procedure has previously been presented in a Figure elsewhere (including test instructions) [35]**,** and the supplementary material from the study protocol entail video exhibitions of the TUGdt test procedure [21].

### Data preparation of TUG-test results

For both TUGdt-NA and TUGdt-MB the number of correct words mentioned during the test performance was documented within the time limit of a finished TUG mobility sequence. This quantification was performed by reviewing the video recordings, following the procedures used in establishing norms for such tests. For TUGdt-NA, both naming an animal group (e.g. fish) and a specific animal (e.g. salmon) were accepted. [36] For TUGdt-MB, the number of correct months in correct order was counted. A month was classified as correct when the participant started with December and then recited months in the correct order relative to the month said previously, with permission to repeat, but not to omit or transpose the months [37]. Dual-task cost was calculated as 100*(TUGdt time score –TUG time score)/TUG time score [38].

Data processing for the step parameters used the video recordings in a semi-automatic method aided by a deep-learning technique for human 2D pose estimation, described in more detail elsewhere [22]. During the extraction of step parameters, the raters were blinded regarding the patients’ diagnoses. By using this technique, the image frames of the gait events heel-strike and toe-off were detected**.** Heel-strike was defined as the first frame after the swing phase where visible contact occurred between foot and floor and the foot was plantarflexed compared to the previous frame. The toe-off was defined as the last frame of stance phase before the toe lost contact with the ground and there was a visible deformation of the shoe’s toe box. Then the rater visually defined the nearest frame for the specific gait event. For the quantification of spatial step parameters, the heel-strike frame was used, where the rater manually marked the most posterior-inferior point of the heel in the side view and the most lateral-inferior point of the heel in the frontal view at heel-strike. The extraction method used for the gait parameters has been validated and tested for inter- and intra-rater reliability with good to excellent results [22].

Quantification of the step parameter outcomes was based on the identified events and positions of the heel points. The 3D positions of the heel points were obtained with the use of a calibration procedure in which known points on the floor were related to image points. *Step length* was calculated as the distance between posterior points of markers on the heel at heel-strike. Since step length is inherently related to body height [39], we divided each participants´ mean step length (centimetres) with their body height (centimetres) to calculate the body/height ratio (%) [40]. Hereafter, step length refers to this ratio. *Step width* was determined by the distance between lateral point of markers of the heels at heel strike. *Step duration*, *single step (SS) duration* and *double stance (DS) duration* were calculated from the times (in seconds) of the identified gait events. Step parameters were analysed for steps starting with the second heel strike after rising from the chair and excluding all steps on the far side of the turning mark at 3 m as well as steps that showed preparations to turn and sit down. For each step parameter, the mean of all analysed steps for each participant was used in the analysis.

TUGdt interference step parameters were calculated as 100 × (TUGdt step parameter value-TUGst step parameter value)/TUGst step parameter value for TUGdt-NA and TUGdt-MB [38].

### Statistical analyses

Participants’ characteristics were summarized using means and standard deviations or frequencies and percentages. The TUG step parameters were not normally distributed and were therefore presented as medians with interquartile ranges and minimum and maximum values. For statistical analyses, TUG step parameters with a Shapiro–Wilk’s test statistic w < 0.95 were transformed using natural logarithms. Using logistic regression models, associations were examined between the various TUG step parameters and the dichotomous variables dementia disorders vs. MCI, MCI vs. SCI, and SCI vs. healthy controls, respectively. In addition, analyses between non-adjacent groups are presented as a supplementary file (Table S1). Results were presented as standardized odds ratios (sORs), i.e. the increase in odds per one standard deviation increase of the TUG step parameter, with 95% confidence intervals (CI^95^) and *p*-values. The logistic regression models were estimated unadjusted and adjusted for participant age, sex, and educational level. The areas under the Receiver Operating Characteristic (ROC) curves (c-statistics) were presented for the step parameters/conditions with the highest sORs and where c-statistics values where ≥ 0.7 which was considered acceptable [41]. Where relevant, optimal cutoff values (based on the point with the highest value for the sum of sensitivity and specificity) were calculated and presented with 95% confidence intervals (CI^95^) based on the bootstrap percentile method. Statistical tests were two-tailed, and the significance level was set at *p* < 0.05. Analyses were carried out using SAS® version 9.4 (SAS Institute Inc., Cary, NC, USA).

## Results

### Participants

Participants (*N* = 304) were divided into the following groups based on their cognitive ability: *controls* (*n* = 50, mean age:73, 44% women, median Verbal Fluency Test score: 22.5), *SCI* (*n* = 71, mean age: 67, 45% women, median Verbal Fluency Test score: 21.0), *MCI* (*n* = 126, mean age: 73, 42% women, median Verbal Fluency Test score: 15.0), and dementia (*n* = 57, mean age:78, 51% women, median Verbal Fluency Test score: 12.0), see Table 1 for full details on participant demographics in the different groups.

**Table 1:** Participant characteristics; TUG & TUGdt test results, and step parameters by group

**Characteristics**	**Dementia** (*N* = 57)^a^		**MCI** (*N* = 126)		**SCI** (*N* = 71)		**Controls** (*N* = 50)	
Age, years, mean ± SD (range)	78 ± 6.6 (55–94)		73 ± 8.6 (50–91)		67 ± 8.9 (39–85)		73 ± 9.3 (50–89)	
Women, n (%)	29 (50.9)		53 (42.1)		32 (45.1)		22 (44.0)	
Married or cohabiting, n (%)	37 (64.9)		84 (66.7)		47 (66.2)		31 (62.0)	
University educated, n (%)	21 (36.8)		52 (41.3)		31 (43.7)		25 (50.0)	
Height (cm), mean ± SD (range)	166.4 ± 8.1 (151–187)		170.3 ± 8.6 (151–193)		171.5 ± 8.9 (153–191)		171.0 ± 9.4 (152–189)	
Verbal Fluency Test, IQR (range)	12.0, 8.3–14.0 (3–28)		15.0, 12.0–19.0 (0–35)		21.0, 17.0–25.0 (11–43)		22.5, 19.0–28.3 (11–37)	
GDS-4, n ≥ 2 (%)	6 (10.6)		25 (19.8)		21 (29.6)		2 (4.0)	
Hand grip (lb), mean ± SD (range)	61.1 ± 22.2 (25–128)		73.8 ± 25.3 (25–130)		82.3 ± 28.5 (34–146)		75.0 ± 25.9 (24–165)	
**TUG Test Results**	*Median (IQR)*	*Range*	*Median (IQR)*	*Range*	*Median (IQR)*	*Range*	*Median (IQR)*	*Range*
TUG (s)	15.1 (13.7–17.6)	7.9–24.1	12.6 (11.1–14.7)	7.4–29.9	10.8 (9.8–12.3)	7.9–26.5	10.4 (9.2–12.1)	7.4–15.1
TUGdt-NA, (s)	18.2 (15.6–20.9)	9.0–34.6	13.9 (11.8–16.7)	7.4–35.4	12.1 (10.6–14.8)	8.0–28.3	12.2 (10.0–15.4)	7.1–22.8
TUGdt-NA (No. named animals)	4 (3–6)	0–10	6 (5–7)	0–10	7 (6–8)	2–12	8 (7–10)	4–15
TUGdt-MB (s)	20.2 (15.5–22.5)	9.8–54.8	14.3 (12.3–18.2)	7.4–44.4	12.3 (10.9–14.5)	8.0–24.2	11.6 (9.9–15.9)	7.6–23.5
TUGdt-MB (No. recited months)	3 (2–4.5)	0–10	6 (4–8)	0–12	8 (6–9)	2–12	10 (8–11)	3–13
**Step Parameter Results**								
**TUG**								
Absolute step length (m)	.48 (.41-.53)	.23-.67	.57 (.50-.63)	.28-.83	.62 (.55-.66)	.37-.85	.6 (.54-.67)	.45-.81
Step length/body height (%)^a^	28.4 (24.9–32.7)	13.741.3	33.3 (29.5–36.3)	17.3–46.8	35.6 (32.8–38.1)	21.0–46.9	35.8 (32.5–38.3)	25.9–45.8
Step width (m)	.19 (.14-.21)	.10-.30	.18 (.15-.21)	.06-.30	.18 (.15-.20)	.08-.26	.17 (.14-.20)	.02-.30
Step duration, (s)	.64 (.60-.68)	.47–1.34	.62 (.57–0.66)	.50-.77	.61 (.57-.64)	.49-.73	.59 (.56-.63)	.48-.68
DS duration, (s)	.15 (0.13-.19)	.01-.61	.13 (.11–0.16)	.06-.22	.13 (.10-.15)	.05-.21	.12 (.09-.15)	.06-.20
SS duration, (s)	.48 (.45-.51)	.39–0.74	.48 (.45–0.51)	.36-.62	.49 (.45-.51)	.4-.59	.48 (.45-.51)	.39-.56
**TUGdt-NA**								
Absolute step length (m)	.45 (.39-.52)	.19-.69	.53 (.46–0.59)	.22–0.78	.59 (.50-.65)	.31-.85	.59 (.52-.63)	.37-.77
Step length/body height (%)^b^	27.4 (24.0–31.2)	11.2–42.1	31.5 (27.6–34.4)	13.8–43.9	33.8 (30.6–36.7)	17.4–46.0	33.7 (30.9–37.6)	23.5–45.0
Step width (m)	.19 (.16-.22)	.06-.33)	.18 (.15–0.21)	.09–0.30	.18 (.14-.20)	.11-.29	.18 (.13-.21)	.07-.31
Step duration (s)	.74 (.67-.80)	.50–1.73	.69 (.62-.75)	.53–1.45	.67 (.62-.72)	.53-.93	.68 (.62-.76)	.51–1.23
DS duration (s)	.20 (.16-.24)	.05–1.07	.16 (.12-.22)	.07-.61	.14 (.12-.18)	.07-.39	.15 (.12-.20)	.07–0.46
SS duration (s)	.54 (.48-.59)	.39–1.10	.52 (.48–0.56)	.42–0.84	.52 (.48-.55)	.43-.72	.54 (.49-.57)	.43-.77
**TUGdt MB**								
Absolute step length (m)	.43 (.36-.51)	.18-.72	.53 (.46-.59)	.19–0.75	.58 (.50-.64)	.34-.80	.60 (.51-.64)	.30-.85
Step length/body height (%)^b^	26.0 (22.9–30.9)	10.9–40.4	31.3 (26.8–34.8)	12.0–41.9	34.3 (29.7–37.0)	19.0–44.9	34.3 (30.1–37.1)	18.4–46.0
Step width (m)	.20 (.16-.23)	.05–0.35	.19 (.16-.22)	.09–0.31	0.16 (0.14–0.21)	.06-.32	.17 (.14-.19)	.05–0.29
Step duration (s)	.74 (.66-.86)	.51–1.86	.71 (.64–0.80)	.53–1.22	0.67 (0.61–0.75)	.51–1.88	.69 (.62-.85)	.50–1.00
DS duration (s)	.22 (.17-.29)	.07–1.13	.17 (.13–0.22)	.06–0.60	0.16 (0.12–0.18)	.08-.86	.15 (.12-.24)	.08–0.35
SS duration (s)	.52 (.46-.58)	.40–1.29	.54 (.49-.59)	.40–0.77	0.52 (0.48–0.57)	.39–1.02	.54 (.49-.62)	.42–0.76

*TUG* The Timed Up and Go test, *TUGdt* TUG with a simultaneous cognitive task, motor cognitive dual-task, *MCI* Mild cognitive impairment, *SCI* Subjective cognitive impairment, *SD* Standard deviation, *cm* centimeters, *GDS-4* Four item version of the Geriatric Depression Scale, *lb* pounds, *IQR* Interquartile range, *s* seconds, *TUGdt-NA* The Timed Up & Go test as a dual-task, naming animals, *TUGdt-MB* The Timed Up & Go test as a dual-task, reciting months in reverse order

^a^Alzheimers disease, *N* = 41, Unspecified dementia, *N* = 11,

^b^Step length/body height ratio = Step length (centimetres) divided with body height (centimetres)

### Performance of the full TUG across groups

The median total time to complete TUG in the different groups were; *control group*: 10.4 s (IQR:9.2–12.1), *SCI group*; 10.8 s (IQR:9.8–12.9), *MCI group*: 12.6 s (IQR:11.1–14.7), and *dementia group*: 15.1 s (IQR:13.7–17.6).

For the *TUGdt-NA* condition, the median total time to complete the test for the different groups were; *control group*: 12.2 s (IQR:10.0–15.4), *SCI group*:12.1 s (IQR:10.6–14.8), *MCI group*:13.9 s, (IQR:10.6–14.8), *dementia group*:18.2 s (IQR:15.6–20.9). For the TUGdt-NA condition, there were small differences in the median number of animals recited between adjacent cognitive ability groups (control = 8, SCI = 7, MCI = 6, dementia = 4).

During the TUGdt-MB condition, the control group completed the task in 11.6 s (IQR: 9.9–15.9) while reciting a median of 10 months correctly. The SCI group completed the task in 12.3 s (IQR: 10.9–14.5) while reciting 8 months correctly. The MCI group completed the TUGdt-MB condition in 14.3 s (IQR: 12.3–18.2) and recited 6 months correctly, while the dementia group completed the task in 20.2 s (IQR: 15.5–22.5) and recited a median of 4 months.

### Comparison of extracted gait parameters between adjacent groups

**Fig. 1:**
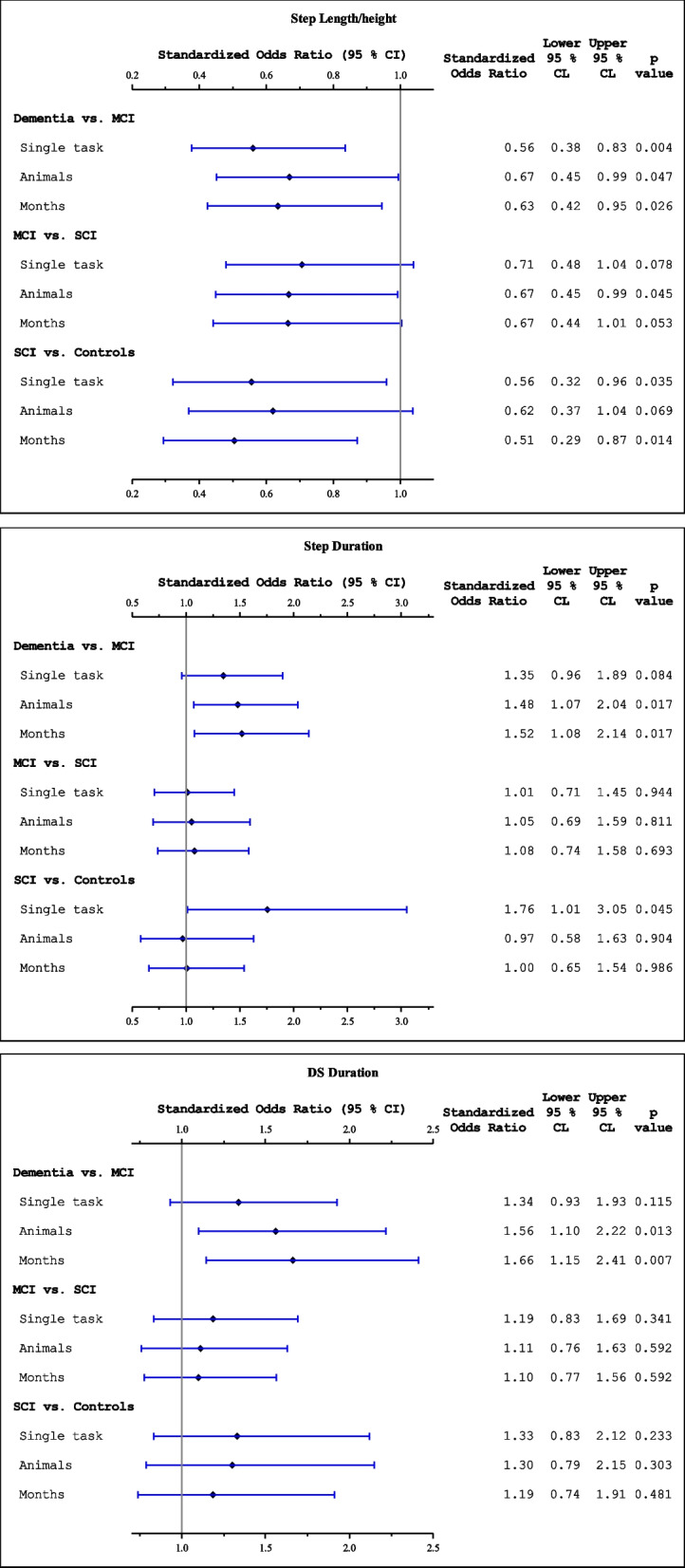
Forest plot of logistic regression models to assess relationship between outcomes dementia disorders vs. MCI, MCI vs. SCI and SCI vs. controls, and TUG step parameters (top panel: step length/ body height; middle panel: step duration; bottom panel: DS duration). Models are adjusted for age, gender and education. Standardized odds ratios measure risk increase per 1 STD increase of the predictor. Statistically significant if *p* < 0.05. MCI = Mild cognitive impairment, SCI = Subjective cognitive impairment

Step length, step duration, SS duration and DS duration were compared between adjacent groups for all three TUG conditions. The median and IQR for all extracted gait parameters are presented in Table 1 and the results for each analysis of step parameter per condition and group are found in Fig. 1 and Table 2 (See Table S1 for comparisons between non-adjacent groups).

**Table 2:** Standardized odds ratios for association between TUG parameters and dementia vs. MCI, MCI vs. SCI and SCI vs. controls

	**Task**	**TUG parameter**	**Unadjusted**		Adjusted*	
			**sOR (CI**^**95**^**)**	***p*-value**	**sOR (CI**^**95**^**)**	***p*-value**
**Dementia vs. MCI**	**TUG**	Step width	.94 (.68–1.30)	.707	.94 (.66–1.33)	.716
		SS duration	1.09 (.82–1.45)	.545	1.11 (.82–1.51)	.506
	**TUGdt-MB**	Step width	1.06 (.76–1.48)	.739	.97 (.67–1.42)	.893
		SS duration	1.13 (.83–1.52)	.441	1.25 (.89–1.75)	.195
		IF step length/body height ratio^a^	.99 (.74–1.33)	.942	1.07 (.79–1.44)	.683
		IF step width	1.17 (.87–1.59)	.303	1.28 (.92–1.78)	.147
		IF step duration	1.42 (1.03–1.95)	**.031**	1.43 (1.02–2.00)	**.039**
		IF SS duration	.96 (.65–1.40)	.819	.88 (.59–1.30)	.512
		IF DS duration	.99 (.74–1.33)	.942	1.07 (.79–1.44)	.683
	**TUGdt-NA**	Step width	1.19 (.86–1.63)	.290	1.22 (.84–1.78)	.301
		SS duration	1.27 (.95–1.68)	.106	1.27 (.94–1.72)	.118
		IF step length/ body height ratio^a^	1.07 (.81–1.42)	.630	1.14 (.85–1.55)	.378
		IF step width	1.28 (.96–1.70)	.094	1.25 (.93–1.69)	.132
		IF step duration	1.42 (1.06–1.90)	**.018**	1.34 (.99–1.81)	.059
		IF SS duration	1.36 (.92–2.00)	.119	1.33 (.89–2.00)	.166
		IF DS duration	1.07 (.81–1.42)	.630	1.14 (.85–1.55)	.378
**MCI vs. SCI**	**TUG**	Step width	1.10 (.81–1.50)	.545	1.03 (0.73–1.46)	.854
		SS duration	.99 (.73–1.35)	.951	.90 (.63–1.27)	.535
	**TUGdt-MB**	Step width	1.59 (1.14–2.21)	**.006**	1.42 (.99–2.04)	.057
		SS duration	1.10 (.76–1.58)	.614	1.02 (.69–1.51)	.909
		IF step length/ body height ratio^a^	.75 (.53–1.06)	.100	.79 (.55–1.15)	.225
		IF step width	1.16 (.78–1.72)	.466	1.11 (.74–1.67)	.602
		IF step duration	1.19 (.80–1.77)	.385	1.09 (.73–1.62)	.677
		IF SS duration	1.91 (1.28–2.85)	**.002**	1.72 (1.14–2.59)	**.009**
		IF DS duration	.75 (.53–1.06)	.100	.79 (.55–1.15)	.225
	**TUGdt-NA**	Step width	1.17 (.86–1.59)	.317	1.07 (.75–1.51)	.718
		SS duration	1.08 (.77–1.53)	.651	.99 (.68–1.44)	.962
		IF step length/ body height ratio^a^	.86 (.63–1.18)	.351	.92 (.66–1.29)	.629
		IF step width	1.16 (.79–1.69)	.444	1.15 (.76–1.73)	.522
		IF step duration	1.16 (.77–1.75)	.475	1.07 (.67–1.69)	.776
		IF SS duration	1.13 (.79–1.62)	.499	1.06 (.73–1.55)	.746
		IF DS duration	.86 (.63–1.18)	.351	.92 (.66–1.29)	.629
**SCI vs. controls**	**TUG**	Step width	1.14 (.80–1.64)	.464	1.23 (0.81–1.86)	.330
		SS duration	1.32 (.86–2.04)	.204	1.44 (0.89–2.33)	.139
	**TUGdt-MB**	Step width	1.02 (.71–1.48)	.905	1.29 (.84–1.96)	.243
		SS duration	.80 (.54–1.19)	.269	.89 (.59–1.35)	.592
		IF step length/ body height ratio^a^	.94 (.59–1.48)	.779	.75 (.46–1.23)	.255
		IF step width	.67 (.45-.99)	**.043**	.75 (.50–1.11)	.145
		IF step duration	.69 (.46–1.04)	.074	.82 (.55–1.22)	.328
		IF SS duration	.84 (0.60–1.17)	.302	.97 (.68–1.39)	.886
		IF DS duration	.94 (0.59–1.48)	.779	.75 (.46–1.23)	.255
	**TUGdt-NA**	Step width	1.21 (.83–1.77)	.314	1.31 (.86–2.02)	.212
		SS duration	.70 (.46–1.08)	.112	.78 (.49–1.23)	.277
		IF step length/ body height ratio^a^	1.19 (.76–1.85)	.446	1.00 (.62–1.61)	.999
		IF step width	.47 (.29-.76)	**.002**	.50 (.30–0.84)	**.008**
		IF step duration	.53 (.32-.89)	**.015**	.61 (.35–1.07)	.084
		IF SS duration	1.01 (.74–1.37)	.952	1.05 (.75–1.45)	.784
		IF DS duration	1.19 (.76–1.85)	.446	1.00 (.62–1.61)	.999

*TUG* Timed Up-and-Go, *MCI* Mild cognitive impairment, *SCI* Subjective cognitive impairment, *sOR* Standardized odds ratios, measure the increase of odds per one standard deviation increase of the TUG parameter, *CI*^*95*^ 95% confidence interval, *SS* Single stance, *DS* Double stance; *TUGdt-MB* Timed Up-and-Go dual-task months backwards, *TUGdt-NA* Timed Up-and-Go dual-task naming animals, *IF* Interference variable of performance of TUGdt compared with TUG, i.e. 100*(TUGdt-TUG)/TUG

^*^adjusted for age, sex, and educational level

^a^step length divided by body height, statistically significant if *p* < 0.05 (indicated in **bold**).

#### Step length

**Fig. 2:**
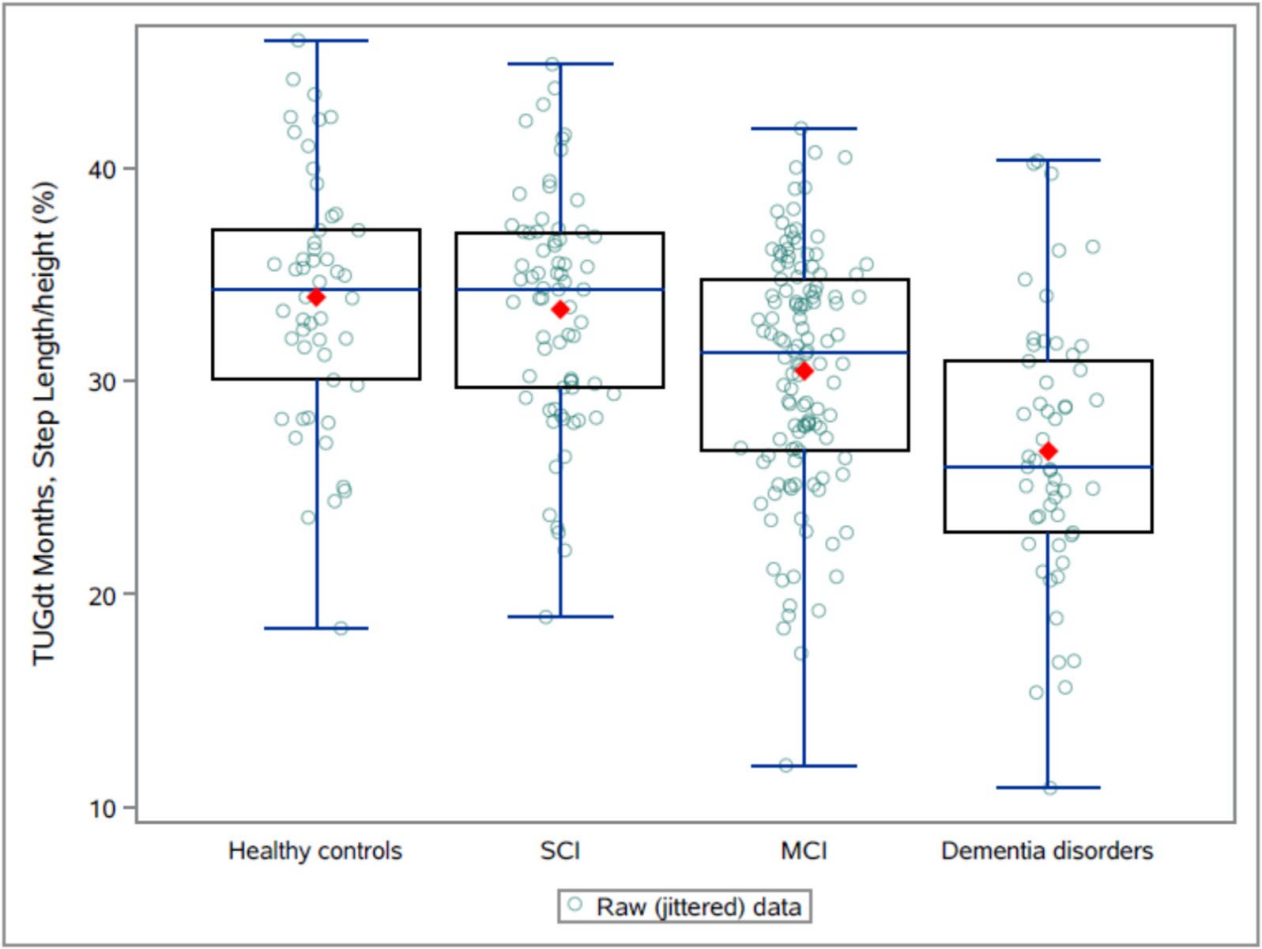
Distribution of results for TUGdt-MB, step length/ body height ratio (%). MCI = Mild cognitive impairment, SCI = Subjective cognitive impairment

The ranges of step length (i.e. the step length/body height ratio) were wide within groups, exemplified by TUGdt-MB (Fig. 2). Nevertheless, the results during TUG showed a trend that the groups with more severe cognitive impairments walked with shorter steps (Table 1). As illustrated in Fig. 1, these results were statistically significant between the SCI group and the control group (sOR = 0.56, CI^95^ = 0.32–0.96), as well as between the dementia group and the MCI group (sOR = 0.56, CI^95^ = 0.38–0.83), but not between the MCI and the SCI group (*p* = 0.078). Similar results were obtained for step length during TUGdt-NA, except that the difference between the SCI group and the control group was not statistically significant (*p* = 0.069). During the TUGdt-MB condition, the results were statistically significant between SCI and controls (sOR = 0.51, CI^95^ = 0.29–0.87), as well as between dementia disorders and MCI (sOR = 0.63, CI^95^ = 0.42–0.95). However, there was no difference between MCI and SCI groups with (*p* = 0.053).

#### Step duration

Comparing step duration for the SCI group and the control group during the TUGst condition (Fig. 1, middle panel) indicated that the SCI group walked with significantly longer step duration (sOR = 1.76, CI^95^ = 1.01—3.05). However, there were no statistically significant differences between the MCI group and the SCI group (*p* = 0.944), nor between the dementia group and the MCI group (*p* = 0.084). During the TUGdt-NA condition there were no statistically significant differences in step duration between the SCI group and the control group (*p* = 0.904), nor between the MCI group and the SCI group (*p* = 0.811). However, the dementia group walked with significantly longer step duration than the MCI group (sOR = 1.48, CI^95^ = 1.07–2.04). Similar results were found during the TUGdt-MB condition where the dementia group had longer step duration than the MCI group (sOR = 1.52, CI^95^ = 1.08–2.14).

#### Double stance duration

Figure 1, bottom panel, presents the DS duration results. For the TUGst condition, there were no significant differences when comparing adjacent cognitive ability groups (p ≥ 0.115). For both TUGdt-NA and TUGdt-MB conditions there were no significant differences in DS duration when comparing the SCI group and the control group or the MCI group and the SCI group (p ≥ 0.303). However, during both TUGdt-NA and TUGdt-MB conditions, the dementia group walked with significantly longer DS duration compared to the MCI group, sOR = 1.56 (CI^95^ = 1.10–2.22) and sOR = 1.66 (CI^95^ = 1.15–2.41), respectively.

#### ROC- curves and optimal cut-off value

**Fig. 3:**
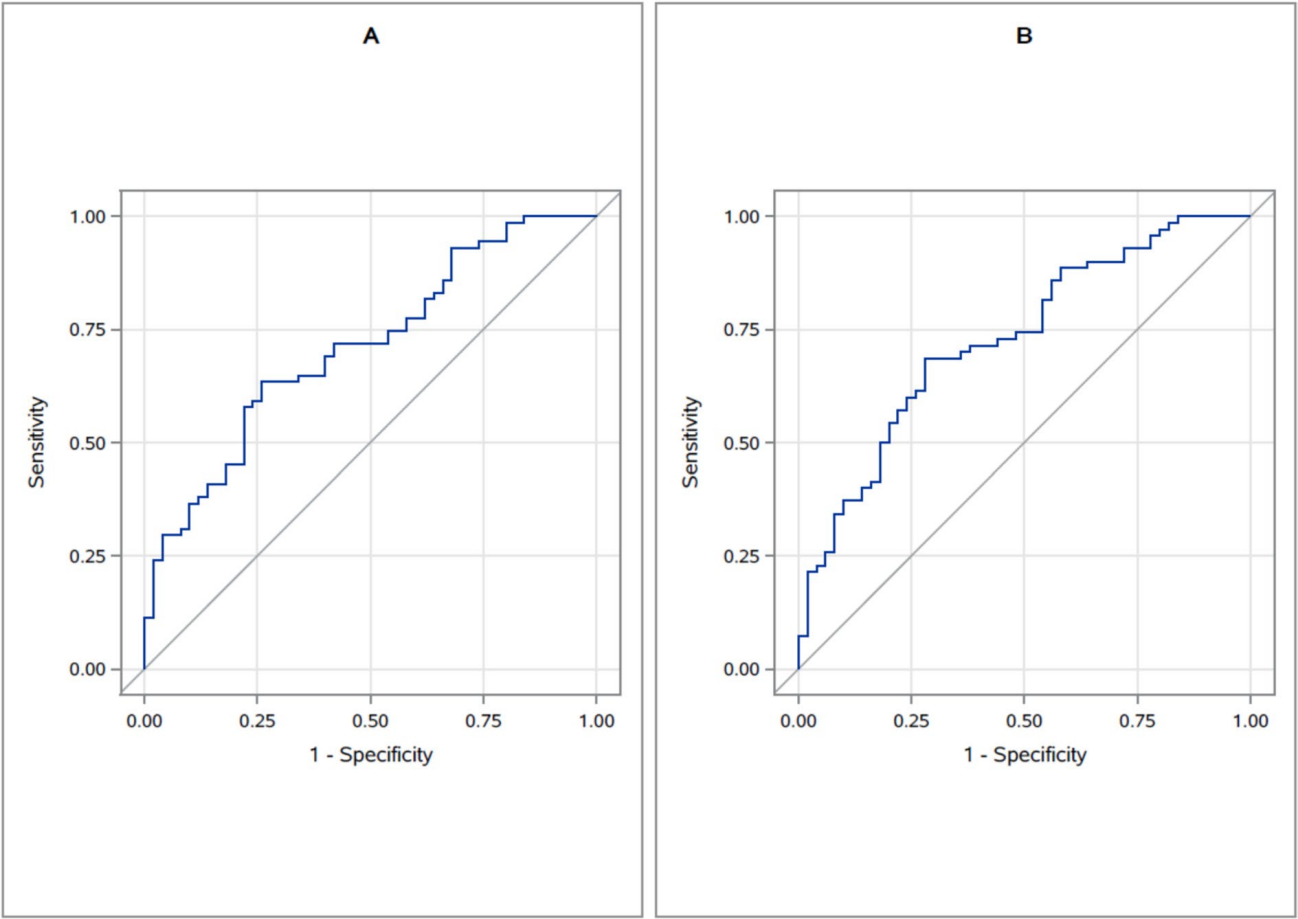
Receiver operating characteristic curves for classification between subjective cognitive impairment and healthy controls based on the step length/body height ratio during A) TUG (C-statistics = .710) and B) TUGdt-MB (C-statistics = .728) and covariates age, sex and education

ROC-curves were conducted for step length since this was the parameter that showed the most potential to discriminate between adjacent groups, with the highest sORs occurring during TUGst and TUGdt-MB for the comparison of the SCI and control groups. As illustrated in Fig. 3, the results showed good predictive ability both for TUGst and TUGdt-MB (C-statistics = 0.729 and 0.700, respectively). The optimal cut-off value was thereafter calculated for the condition with the highest sOR. These results showed that using the step-to height ratio, a ratio of less than 32.9% (CI^95^ = 22.1–43.0) while conducting the TUGdt-MB indicated that an individual experienced SCI.

## Discussion

This is the first study to investigate if specific step parameters during the TUG test can discriminate between groups with different cognitive ability. The results varied depending on group comparison, TUG condition and step parameter. Nevertheless, step length was the step parameter that most consistently discriminated between the different groups and gait conditions.

Considering the step length/height parameter, both the TUG and TUGdt-MB conditions significantly discriminated between the groups, with the highest sORs occurring during the latter condition. Conversely, TUGdt-NA was the only condition that significantly discriminated between the MCI and the SCI groups, whereas all TUG conditions discriminated between the dementia and MCI groups (with the highest sOR for the TUG condition). For step duration, only the TUG condition significantly discriminated between the SCI and control groups, whereas both TUGdt conditions significantly discriminated between the dementia and MCI groups, with similar sORs. The only groups that DS duration significantly discriminated between were the dementia and MCI groups, and only during the TUGdt conditions, where TUGdt-NA had the highest sOR. Since our results suggested that step length was the parameter that showed the highest potential to discriminate between adjacent groups, ROC curves were conducted for the conditions that showed the highest sORs (i.e. TUGst and TUGdt-MB for the comparison of SCI and control groups). Since the results showed acceptable predictive ability (C ≥ 0.7) for both these conditions, the optimal cut-off value (using the established step length to body-height ratio) was then calculated for TUGdt-MB, as it had the highest sOR of the two conditions. A step length of less than 32.9% of the body height while conducting the TUGdt-MB was the optimal cut-off value for indicating that an individual experienced SCI.

With some variation, there was an overall trend that groups with more severe cognitive impairment walked with shorter steps both during the TUG condition and the two TUGdt conditions. These results are in accordance with previous studies where shorter stride/step length has been found to occur in groups with dementia compared to MCI and/or controls [10, 42] as well as between people with MCI and controls [43]. However, those studies were conducted while participants performed straight, overground walking, primarily in movement laboratory settings. The finding that the group with SCI walked with shorter steps than controls is a particularly novel and potentially important finding, rarely identified in previous studies [44]. This is of particular interest as SCI is defined as “self-reported impairment that cannot be identified during standard cognitive assessment”. Bearing in mind the importance of early detection of cognitive impairment, this result may highlight the importance of investigating step length during the TUG test during both single and dual-task conditions. Therefore, the optimal cut-off calculated in this study may be used to obtain an absolute value when assessing patients in clinical settings. Nevertheless, this is a novel finding that needs to be confirmed by future studies.

This study was designed to be conducted in the clinical settings of two different hospitals, where individuals undergoing memory assessment were recruited. This was done to maximize the study´s ecological validity as well as to facilitate integrating this assessment into a standard cognitive assessment battery, should the results motivate this. Given that it has previously been highlighted that deviant gait precedes cognitive impairment, and that such deviances may be even more evident while conducting motor-cognitive dual-tasking, two different types of cognitive task were integrated into the protocol. The first task, TUGdt-NA, is an established dual-task [45] inspired by the verbal fluency test. The other task, TUGdt-MB, was first presented in a pilot study from our research group [21]. This task was developed to be a more feasible version of the common task counting backwards by 7 s. Indeed, the importance of using a cognitive task of adequate difficulty (i.e. not too easy nor too difficult) has been emphasized in the developing field of motor-cognitive dual-tasking [46]. Since the aim of UDDGait is to pin-point individuals that already experience cognitive deficits, this task, developed to challenge cognitive inhibition, was considered of appropriate difficulty level while also being feasible to use during clinical assessment. Regardless of the step parameter considered, no TUG condition significantly discriminated between all adjacent groups. However, the only non-significant discrimination by step length for the TUGdt-MB condition was between the MCI and SCI groups. These findings may indicate that the TUGdt-MB task is preferable to TUGdt-NA for discriminating between groups with low levels of cognitive impairment, which might be argued to be of particular importance for early detection of cognitive decline. The interpretation of the potential advantage of TUGdt-MB is also supported by the findings in a previous study by our research group, where this TUG-condition had the highest OR when using the parameter *number of months correctly recited per 10 s* for discriminating between SCI and controls [23].

This study has several limitations. Since participants only conducted one trial per gait condition, the total number of steps included to calculate parameter mean values was limited which may be a reason for the high variability found within the different parameters. This is one reason why previous studies using an instrumented TUG have often extended the distance [47], which in turn require more spacious assessment facilities. Another potential limitation is that body height rather than leg length was recorded since this information could be derived from patient charts. Although step length is proportional both to leg length and body height [48], the lack of a direct measure of leg length may interfere with the specificity of these results. On the other hand, this project was designed to facilitate the implementation of relevant findings into clinical practice. Therefore, the assessment procedures were conducted similar to how patients are assessed in healthcare settings (i.e. conducting each test once, not least due to time constraints). In addition, participants were older adults with varied cognitive and physical status, therefore using only one trial per TUG condition minimized fatigue in those participants with less physical or cognitive reserves. Another practical decision to reduce the risk of cognitive fatigue among the participants was to not assess the cognitive tasks used during the TUGdt conditions as single tasks due to the extensive test battery. This limited the ability to assess potential task prioritization. However, bearing in mind that clinical research has been criticized for commonly excluding potential participants with higher degrees of disability [49], certain trade-offs may be required to enable inclusive clinical trials. Another limitation of this study was the use of multiple analyses, which may result in significant results by chance. One way to reduce this possibility would have been to use the Bonferroni correction. However, the Bonferroni correction is highly conservative which can lead to a substantial risk of type 2 error [50]. Instead, these results should be interpreted with caution and confirmed by future research.

## Conclusions

The results of this study indicated that step length during the clinically applicable test conditions TUG and TUGdt-MB discriminated between groups with different cognitive ability, where shorter step length, particularly during TUGdt-MB, was related to more profound impairment. In addition, this is the first study to present a clinically applicable cut-off to indicate cognitive impairment in groups with milder cognitive impairment. Although these findings need to be confirmed by future studies, they highlight the potential of using step parameters during different TUG-conditions for early detection of cognitive impairment.

## Supplementary Information

Supplementary Material 1.

## Data Availability

The material analysed during the current study is not publicly available due to its content of sensitive personal data. Datasets generated may be available from the principal investigator (ACÅ) on reasonable request, after ethical considerations.

## Abbreviations

CI^95^: 95% Confidence Interval
DS: Double Stance
MCI: Mild Cognitive Impairment
OR: Odds Ratio
ROC: Receiver Operator Characteristic
SCI: Subjective Cognitive Impairment
sOR: Standardized Odds Ratio
SS: Single Stance
TUG: Timed Up and Go
TUGdt-NA: Timed Up and Go as a motor-cognitive dual-task with the secondary task entailing naming animals
TUGdt-MB: Timed Up and Go as a motor-cognitive dual-task with the secondary task entailing reciting months in reverse order
