# Supplementary material for: Extracted step parameters during the timed up and go test discriminate between groups with different levels of cognitive ability—a cross-sectional study
Source: BMC Geriatr. 2025 Mar 17;25:182. doi: 10.1186/s12877-025-05828-6 (PMC11912623; doi:10.1186/s12877-025-05828-6)
Supplement: Supplementary file 1 — Supplementary Material 1. [file 12877_2025_5828_MOESM1_ESM.docx]

|  | | | | | | |
| --- | --- | --- | --- | --- | --- | --- |
| **Table S1.** Standardized odds ratios for association between TUG parameters and dementia vs. controls, dementia vs. SCI, and MCI vs. controls | | | | | | |
|  | **Task** | **TUG parameter** | **Unadjusted** | | **Adjusted*** | |
|  |  |  | **sOR (CI^95^)** | **p-value** | **sOR (CI^95^)** | **p-value** |
| **Dementia vs.controls** | **TUG** | Step length/body height ratio^1^ | .24 (.13-.41) | **<.001** | .23 (.12-.44) | **<.001** |
|  |  | Step width | 1.15 (.81-1.65) | .431 | 1.27 (0.84-1.91) | .255 |
|  |  | Step duration | 2.23 (1.36-3.66) | **.001** | 2.27 (1.31-3.95) | **.004** |
|  |  | SS duration | 1.29 (.89-1.87) | .173 | 1.34 (0.87-2.08) | .187 |
|  |  | DS duration | 2.03 (1.28-3.23) | **.003** | 1.73 (1.08-2.79) | **.024** |
|  | **TUGdt-MB** | Step length/height | 0.30 (0.18-0.50) | **<.001** | .29 (0.16-0.53) | **<.001** |
|  |  | Step width | 1.59 (1.06-2.38) | **.024** | 1.66 (1.06-2.62) | **.028** |
|  |  | Step duration | 1.37 (0.97-1.93) | .074 | 1.31 (0.91-1.88) | .140 |
|  |  | SS duration | 1.00 (0.74-1.36) | .999 | 1.05 (0.75-1.48) | .767 |
|  |  | DS duration | 2.04 (1.32-3.17) | **.001** | 1.75 (1.10-2.79) | **.019** |
|  |  | IF step length/ body height ratio^1^ | 0.82 (0.58-1.15) | .254 | .90 (0.63-1.28) | .552 |
|  |  | IF step width | 0.93 (0.69-1.26) | .643 | .97 (0.70-1.34) | .861 |
|  |  | IF step duration | 1.10 (0.80-1.50) | .555 | 1.07 (0.77-1.49) | .676 |
|  |  | IF SS duration | 1.17 (0.83-1.66) | .370 | 1.05 (0.71-1.54) | .805 |
|  |  | IF DS duration | 0.82 (0.58-1.15) | .254 | .90 (0.63-1.28) | .552 |
|  | **TUGdt-NA** | Step length/ body height ratio^1^ | 0.33 (0.20-0.54) | **<.001** | 0.35 (0.20-0.61) | **<.001** |
|  |  | Step width | 1.60 (1.08-2.38) | **.019** | 1.88 (1.17-3.02) | **.009** |
|  |  | Step duration | 1.47 (1.03-2.11) | **.035** | 1.35 (0.93-1.97) | .112 |
|  |  | SS duration | 1.06 (0.77-1.46) | .712 | 1.07 (0.76-1.51) | .694 |
|  |  | DS duration | 2.02 (1.29-3.15) | **.002** | 1.70 (1.08-2.67) | **.022** |
|  |  | IF step length/ body height ratio^1^ | 1.06 (0.74-1.51) | .744 | 1.17 (0.80-1.70) | .420 |
|  |  | IF step width | 0.92 (0.68-1.25) | .613 | 0.94 (0.68-1.29) | .683 |
|  |  | IF step duration | 1.09 (0.81-1.47) | .558 | 1.04 (0.76-1.41) | .818 |
|  |  | IF SS duration | 1.23 (0.89-1.70) | .212 | 1.18 (0.83-1.68) | .363 |
|  |  | IF DS duration | 1.06 (0.74-1.51) | .744 | 1.17 (0.80-1.70) | .420 |
| **Dementia vs. SCI** | **TUG** | Step length/ body height ratio^1^ | 0.26 (0.16-0.43) | **<.001** | 0.40 (0.22-0.72) | **.002** |
|  |  | Step width | 1.03 (0.71-1.50) | .872 | 1.04 (0.66-1.66) | .853 |
|  |  | Step duration | 1.75 (1.16-2.62) | **.007** | 1.64 (0.98-2.75) | .060 |
|  |  | SS duration | 1.09 (0.79-1.50) | .616 | 1.10 (0.70-1.75) | .673 |
|  |  | DS duration | 2.00 (1.31-3.07) | **.001** | 1.61 (1.06-2.43) | **.025** |
|  | **TUGdt-MB** | Step length/ body height ratio^1^ | 0.29 (0.18-0.48) | **<.001** | 0.47 (0.26-0.83) | **.009** |
|  |  | Step width | 1.56 (1.08-2.25) | **.019** | 1.38 (0.88-2.16) | .164 |
|  |  | Step duration | 1.62 (1.14-2.32) | **.007** | 1.74 (1.14-2.64) | **.010** |
|  |  | SS duration | 1.14 (0.85-1.53) | .386 | 1.52 (1.01-2.28) | **.046** |
|  |  | DS duration | 2.50 (1.58-3.95) | **<.001** | 1.93 (1.20-3.11) | **.007** |
|  |  | IF step length/ body height ratio^1^ | 0.81 (0.56-1.16) | .240 | 1.02 (0.69-1.51) | .913 |
|  |  | IF step width | 1.20 (0.88-1.63) | .246 | 1.57 (1.05-2.35) | **.029** |
|  |  | IF step duration | 1.42 (1.01-2.00) | **.042** | 1.55 (1.05-2.28) | **.029** |
|  |  | IF SS duration | 1.55 (1.03-2.33) | **.034** | 1.28 (0.81-2.02) | .299 |
|  |  | IF DS duration | 0.81 (0.56-1.16) | .240 | 1.02 (0.69-1.51) | .913 |
|  | **TUGdt-NA** | Step length/ body height ratio^1^ | 0.34 (0.22-0.53) | **<.001** | 0.57 (0.33-1.00) | **.048** |
|  |  | Step width | 1.42 (0.97-2.09) | .070 | 1.52 (0.90-2.54) | .116 |
|  |  | Step duration | 1.99 (1.30-3.05) | **.002** | 1.72 (1.05-2.80) | **.030** |
|  |  | SS duration | 1.31 (0.95-1.80) | .101 | 1.47 (0.96-2.26) | .078 |
|  |  | DS duration | 2.53 (1.57-4.07) | **<.001** | 1.75 (1.05-2.90) | **.031** |
|  |  | IF step length/ body height ratio^1^ | 0.96 (0.68-1.34) | .797 | 1.36 (0.90-2.06) | .148 |
|  |  | IF step width | 1.43 (0.99-2.07) | .059 | 1.48 (0.95-2.30) | .082 |
|  |  | IF step duration | 1.61 (1.08-2.40) | **.019** | 1.40 (0.90-2.16) | .134 |
|  |  | IF SS duration | 1.46 (0.97-2.19) | .072 | 1.44 (0.87-2.37) | .151 |
|  |  | IF DS duration | 0.96 (0.68-1.34) | .797 | 1.36 (0.90-2.06) | .148 |
| **MCI vs. controls** | **TUG** | Step length/ body height ratio^1^ | 0.51 (0.34-0.77) | **.001** | 0.42 (0.26-0.68) | **<.001** |
|  |  | Step width | 1.23 (0.89-1.70) | .203 | 1.27 (0.90-1.80) | .180 |
|  |  | Step duration | 1.50 (1.03-2.18) | **.036** | 1.59 (1.05-2.40) | **.027** |
|  |  | SS duration | 1.25 (0.87-1.79) | .221 | 1.25 (0.86-1.80) | .242 |
|  |  | DS duration | 1.49 (1.02-2.16) | **.039** | 1.60 (1.06-2.42) | **.024** |
|  | **TUGdt-MB** | Step length/ body height ratio^1^ | 0.49 (0.33-0.74) | **<.001** | 0.39 (0.24-0.63) | **<.001** |
|  |  | Step width | 1.64 (1.13-2.39) | **.009** | 1.90 (1.24-2.91) | **.003** |
|  |  | Step duration | 0.96 (0.65-1.43) | .857 | 0.98 (0.65-1.50) | .941 |
|  |  | SS duration | 0.82 (0.54-1.23) | .332 | 0.83 (0.55-1.25) | .374 |
|  |  | DS duration | 1.11 (0.78-1.58) | .567 | 1.16 (0.78-1.71) | .466 |
|  |  | IF step length/ body height ratio^1^ | 0.74 (0.52-1.07) | .106 | 0.72 (0.50-1.04) | .082 |
|  |  | IF step width | 0.66 (0.45-0.98) | **.038** | 0.67 (0.45-1.00) | .052 |
|  |  | IF step duration | 0.71 (0.47-1.05) | .088 | 0.70 (0.45-1.07) | .099 |
|  |  | IF SS duration | 1.34 (0.91-1.98) | .136 | 1.39 (0.93-2.08) | .110 |
|  |  | IF DS duration | 0.74 (0.52-1.07) | .106 | 0.72 (0.50-1.04) | .082 |
|  | **TUGdt-NA** | Step length/ body height ratio^1^ | 0.53 (0.35-0.79) | **.002** | 0.44 (0.28-0.71) | **<.001** |
|  |  | Step width | 1.38 (0.99-1.94) | .059 | 1.51 (1.03-2.21) | **.033** |
|  |  | Step duration | 0.96 (0.65-1.40) | .815 | 0.96 (0.65-1.42) | .833 |
|  |  | SS duration | 0.78 (0.54-1.12) | .183 | 0.77 (0.53-1.12) | .174 |
|  |  | DS duration | 1.20 (0.83-1.74) | .337 | 1.25 (0.84-1.87) | .269 |
|  |  | IF step length/ body height ratio^1^ | 0.98 (0.70-1.38) | .921 | 0.95 (0.67-1.34) | .778 |
|  |  | IF step width | 0.61 (0.43-0.88) | **.009** | 0.60 (0.41-0.88) | **.009** |
|  |  | IF step duration | 0.69 (0.47-0.99) | **.046** | 0.66 (0.45-0.99) | **.042** |
|  |  | IF SS duration | 1.09 (0.79-1.50) | .588 | 1.10 (0.79-1.52) | .569 |
|  |  | IF DS duration | 0.98 (0.70-1.38) | .921 | 0.95 (0.67-1.34) | .778 |
| *****=adjusted for age, sex, and educational level, ^1^= step length divided by body height, statistically significant if p < 0.05 (indicated in **bold**).  TUG = Timed Up-and-Go, MCI=Mild cognitive impairment, SCI= Subjective cognitive impairment, sOR = Standardized odds ratios, measure the increase of odds per one standard deviation increase of the TUG parameter, CI^95^ = 95% confidence interval, SS = single stance; DS = double stance; TUGdt MB = Timed Up-and-Go dual-task, reciting months in reverse order;TUGdt NA = Timed Up-and-Go dual-task naming animals, IF = interference variable of performance of TUGdt compared with TUG, i.e. 100*(TUGdt-TUG)/TUG | | | | | | |
